# SENSE‐Cog Residential Care: A Pilot Cluster Randomized Trial on Hearing and Vision Support for Residents with Dementia in Long‐term Care

**DOI:** 10.1002/alz70858_098696

**Published:** 2025-12-25

**Authors:** Iracema Leroi

**Affiliations:** ^1^ Trinity College Dublin, Dublin, Ireland

## Abstract

**Background:**

Hearing and vision loss are common among residents with dementia (RwD) in long‐term care (LTC) facilities, adversely affecting quality of life and dementia‐related outcomes. Enhancing sensory function may improve these outcomes. The SENSE‐Cog Residential Care pilot trial evaluated the feasibility of a comprehensive sensory intervention for RwD with hearing and vision loss and explored the potential for a definitive evaluation of sensory support programs in LTC.

**Method:**

This pilot, feasibility, cluster‐randomized controlled trial included nine LTC facilities in Ireland. Facilities were assigned to either “care as usual” (CAU) or a multi‐component sensory intervention. The intervention comprised personalized hearing and vision support, staff training on sensory health, creation of a sensory‐friendly environment, and mapping of sensory care provision. The trial assessed feasibility, acceptability, and tolerability among residents and staff, with exploratory outcomes for a future definitive trial. The primary goal was to inform the design of a large‐scale trial. Data were collected on recruitment and retention rates, intervention uptake, safety, and data collection feasibility. Additional measures included the provision of hearing and vision assessments and devices, quality of life as an exploratory outcome, and staff feedback on training.

**Results:**

Nine LTC facilities consented to participate. Of 42 RwD screened, 27 were recruited (13.5/month), with 26 retained at three months. In intervention facilities, all 12 residents received hearing and vision assessments, resulting in provision of glasses (12), hearing aids (4), and listening devices (6). Sensory‐cognitive training was delivered to 42 staff, including in‐depth sensory champion training for two staff per facility. Staff rated the training highly (TARS acceptability: *M* = 33.18; outcomes: *M* = 30). Environmental audits highlighted limited capacity for structural sensory changes. Data collection compliance exceeded 95%, with 75% of RwD wearing glasses, 50% using hearing aids, and 33% utilizing listening devices at follow‐up. No safety concerns were reported.

**Conclusion:**

Recruitment, intervention uptake, and data collection in LTC facilities were feasible, and staff and resident participation was high. These findings support a full‐scale randomized controlled trial to evaluate the efficacy of sensory interventions in LTC settings.

